# Diverse clinical manifestations of Parvovirus B19 infections during the 2024 outbreak in Germany

**DOI:** 10.1007/s15010-025-02598-6

**Published:** 2025-07-16

**Authors:** A. Holzem, J. Stemler, S. Böhm, H. Gruell, A. Zeuzem, E. Schalk, T. Schober, C. Deppe, J. Hübner, M. von Bergwelt-Baildon, K. Spiekermann, for the Infectious Diseases Working Party (AGIHO) of the German Society of Hematology and Medical Oncology (DGHO)

**Affiliations:** 1 Department of Medicine III, LMU University Hospital, Munich, Germany; 2 Faculty of Medicine and University Hospital Cologne, Institute for Translational Research, Cologne Excellence Cluster on Cellular Stress Responses in Aging- Associated Diseases (CECAD), University of Cologne, Cologne, Germany; 3 Faculty of Medicine, Department I of Internal Medicine, Center for Integrated Oncology Aachen Bonn Cologne Duesseldorf (CIO ABCD) and Excellence Center for Medical Mycology (ECMM), University of Cologne, University Hospital Cologne, Cologne, Germany; 4 German Centre for Infection Research (DZIF), Partner Site Bonn-Cologne, Cologne, Germany; 5 National Reference Center for Retroviruses, Faculty of Medicine, Max von Pettenkofer-Institute, LMU Munich, Virology, Germany; 6 Institute of Virology, Faculty of Medicine and University Hospital Cologne, University of Cologne, Cologne, Germany; 7 Department of Hematology, Oncology and Cell Therapy, Medical Faculty, Otto von Guericke University, Magdeburg, Magdeburg, Germany; 8 Division Pediatric Infectious Diseases, Dr. von Hauner Children’s Hospital, LMU University Hospital, Munich, Germany; 9 Department of Gynecology and Obstetrics, LMU University Hospital, Munich, Germany

**Keywords:** Parvovirus B19, Germany, Outbreak, Laboratory surveillance

## Abstract

**Purpose:**

In 2024, human parvovirus B19 (PB19V) infections have increased in Germany and globally. It is an infection associated with a broad spectrum of clinical manifestations. To raise awareness, we present representative cases and virological data from different specialties across three German university hospitals.

**Methods:**

Following a nationwide survey by the AGIHO in March 2024 indicating increased PB19V infections, we conducted a retrospective, multi-center descriptive study across Munich, Cologne, and Magdeburg. Anonymized clinical and virological data from 2022 to 2024 were collected, including patient demographics, underlying diseases, and diagnostic findings. Acute PB19V infection was defined by real-time quantitative PCR-based detection of PB19V DNA in any specimen.

**Results:**

Clinical manifestations of acute PB19V infections can range from severe anemia and pancytopenia in hematologic patients, to fetal hydrops in pregnant women, and systemic inflammatory symptoms in patients with chronic conditions. In 2024, the Max von Pettenkofer Institute in Munich conducted 936 PB19V PCR tests. A marked increase in positive cases was observed in early 2024, with positivity rates of 16% in Q1 and 18.2% in Q2, compared to an annual positivity rate of 2.3% in 2023. Similar trends were seen at the University Hospitals Cologne and Magdeburg. Most infections were acute with high viral loads. Most cases originated from pediatric, gynecologic, and hematologic departments, highlighting particularly vulnerable patient populations.

**Conclusions:**

This resurgence in symptomatic PB19V infections, likely driven by pandemic-related shifts in immunity and exposure, underscores the need for heightened clinical awareness, early testing in high-risk populations, and sustained surveillance to anticipate future outbreaks.

## Introduction

**Fig. 1:**
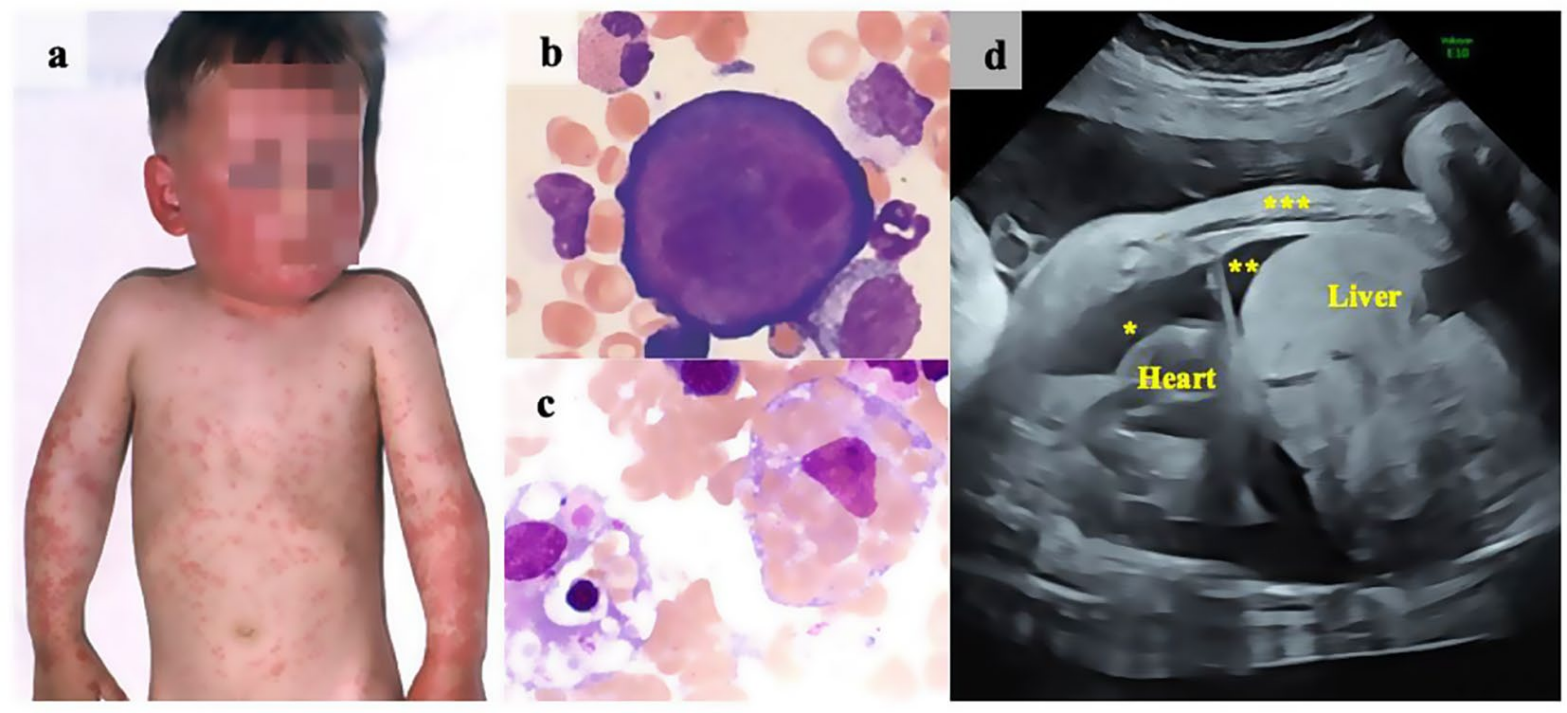
**a**) Characteristic rash of erythema infectiosum (“slapped cheek”) **b**) Bone marrow smear showing a proerythroblast with intranuclear inclusion bodies as hallmark of PB19V infection in erythroid progenitor cells **c**) Bone marrow smear with a macrophage engulfing hematopoietic cells, indicative of hemophagocytosis (b, c: May-Grünwald-Giemsa stain, 1000x) **d**) Fetal ultrasound showing pleural effusion*, ascites**, and skin edema*** as sign of hydrops fetalis

Human parvovirus B19 (PB19V) is a non-enveloped, single-stranded DNA virus that targets erythroid progenitor cells leading to transient erythropoietic arrest. PB19V can cause a range of symptoms that vary depending on the patient’s age and immune status (Fig. 1 Overview of clinical manifestations associated with PB19V infection). In children, primary infection often causes erythema infectiosum with a facial rash and flu-like symptoms (Fig. 1a). Adults more commonly present with joint pain and swelling, often without the rash. Bone marrow findings include giant proerythroblasts with intranuclear inclusions where viral replication takes place (Fig. 1b). Severe complications, though rare, include myocarditis, encephalitis, and hemophagocytic lymphohistiocytosis (HLH) [1]. In cases of HLH, the bone marrow shows hemophagocytosis with activated macrophages destroying hematopoietic cells (Fig. 1c). During pregnancy, vertical transmission may result in fetal anemia, hydrops fetalis and even intrauterine fetal death (IFD). Malformations due to a PB19V infection are not known [2, 3]. With doppler sonography signs of hydrops fetalis such as pleural effusion, ascites, skin edema, and widened nuchal translucency (Fig. 1d), are detectable. Immunocompromised patients, such as those with hematologic comorbidities or following stem cell transplantation, are at risk of severe and acute anemia also referred to as aplastic crisis, or may develop chronic courses with pure red cell aplasia. PB19V is typically transmitted via respiratory droplets. In acutely infected individuals, the virus can be detected in the blood with a high viral load (> 10^4^ copies/mL). Diagnosis is achieved through the detection of viral DNA via polymerase chain reaction (PCR) in serum, bone marrow, biopsy tissue, and other materials. IgG and IgM antibodies, along with IgG avidity testing help differentiate acute and past infection. By age 15 years, around 50% of individuals have been exposed to the virus, and by age 70 years, seroprevalence reaches 80–100% [4]. Currently, there are no antiviral agents or vaccines available, and management focuses on symptomatic treatment, including transfusions and immunoglobulin (IVIG) therapy in immunocompromised patients [5]. In 2024, multiple centers across Germany reported a rising number of PB19V infections, prompting this brief report to highlight the diverse clinical presentation. In the absence of a national reporting system, we supplemented our analysis with virological data from participating institutions.

## Methods

In March 2024, the Infectious Diseases Working Party (AGIHO) of the German Society for Hematology and Medical Oncology (DGHO) conducted a national survey revealing an increased incidence of PB19V infections across centers. In response, we initiated a retrospective, multi-center, descriptive study of infected patients in Munich, Cologne, and Magdeburg. This report presents one case in detail, including laboratory results throughout the hospital stay, and summarizes additional cases from two institutions across three clinical departments. Anonymized clinical data were collected, including patient demographics, underlying conditions, hemoglobin levels at admission, and other relevant findings. Acute PB19V infection was defined by the detection of PB19V DNA via real-time quantitative PCR (qPCR). In Magdeburg, testing was performed with the RealStar Parvovirus B19 PCR Kit 1.5 (Altona Diagnostics, Cat. No. AS0101543), and in Cologne with the RealStar Parvovirus B19 PCR Kit 1.0 (Altona Diagnostics, Cat. No. 101013); both are CE-IVD-certified commercial kits with predefined primers, probes, and reagents. In Munich, an in-house qPCR assay targeting the NS1 gene (161 bp, nt 2083–2243; NCBI RefSeq NC_000883) was used, with amplification performed using the TaqMan Universal PCR Master Mix (Applied Biosystems). Virological data from all three sites, spanning from 2022 to the end of 2024, were included. Results were reported as genome equivalents per milliliter (Geq/mL) or international units per milliliter (IU/mL), as applicable. Materials tested included serum, saliva, bone marrow, biopsy tissue, and cerebrospinal fluid.

### Case presentations

**Fig. 2:**
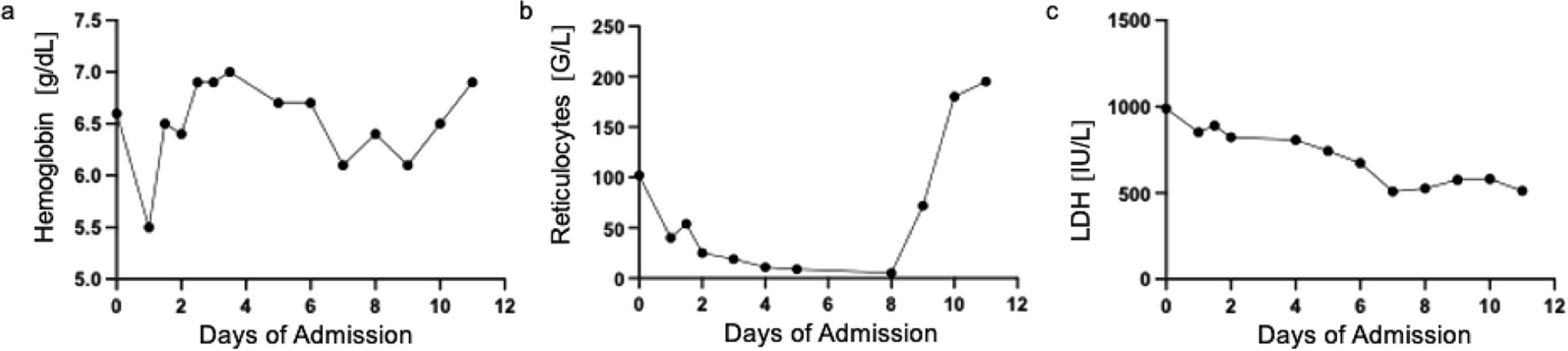
Line graphs illustrating **a**) hemoglobin (g/dl), **b**) reticulocyte count, and **c**) lactate dehydrogenase (LDH) (U/l) of the presented case throughout the hospital stay of 12 days

A patient in their 30s presented to the emergency department of LMU University Hospital with general debilitation, fever (up to 39.4 °C), and joint pain. On physical examination, dyspnea and splenomegaly (17.5 × 5.5 cm) were noted. The medical history included heterozygous hereditary spherocytosis (HS). Laboratory tests revealed hemolytic anemia (Hb 6.6 g/dL, bilirubin 8.6 mg/dL, LDH 988 U/L, reticulocytes 102‰, haptoglobin < 0.1 g/L), a CRP of 8.2 mg/L, and a negative Coombs test. Chest X-ray and blood cultures showed no infection. Microbiological testing confirmed a positive PCR result for PB19V, with a viral load exceeding 10^8^ Geq/mL, while serology was initially negative. An aplastic crisis was confirmed by a reticulocyte production index (RPI) < 0.10, and six RBC units were transfused in the first three days. IVIG was not administered. In addition, E. coli was detected in the patient’s urine, leading to a presumptive diagnosis of a urinary tract infection. The patient was treated symptomatically with paracetamol and empiric antibiotics. By day nine post-admission, seroconversion occurred, with both PB19V-IgM and -IgG antibodies turning positive. Two weeks after discharge, her viral load had decreased to 18,000 Geq/mL, and all clinical symptoms had resolved. An RPI of 3.69 indicated active erythropoiesis, with no signs of anemia. Figure 2 (Fig. 2 Laboratory parameters throughout the hospital stay) illustrates the progression of key laboratory parameters during her hospital stay.

### Facets of PB19V infection

By correlating clinical severity, hematologic changes, and timing of symptoms with virological findings, we aim to contextualize the subsequent virological analyses and emphasize their clinical relevance. The detailed case (Fig. 2) illustrates the progression of key laboratory and clinical parameters during hospitalization. The summary of additional cases (Tables 1 and 2) captures the broader clinical spectrum of PB19V infection across centers.

In Munich, we observed several severe PB19V cases with heterogeneous presentations (Table 1, Munich_Hem). A patient in their 20s (Munich_Hem1) with HS presented with symptomatic anemia (Hb 5.9 g/dL), requiring four RBC transfusions. The PB19V viral load exceeded 100 million Geq/mL Over the course, the patient developed hepatitis, arthritis, and gastroenteritis, complicating management. In contrast, a teenage patient (Munich_Hem2) also with HS experienced an aplastic crisis with similar hemoglobin levels (5.9 g/dL) but had no inflammatory signs or complications. The patient received two RBC units and was managed as an outpatient. This illustrates that PB19V infection in predisposed individuals can range from mild to severe symptoms. A patient in their 40s (Munich_Hem3) was referred to LMU University Hospital with an acute PB19V infection and a viral load exceeding 100 million Geq/mL, with seronegative status. The case was further complicated by PB19V-triggered hemophagocytic lymphohistiocytosis (HLH), fulfilling 7 of 9 HLH-2004 diagnostic criteria [6]. These included fever, splenomegaly, pancytopenia, hyperferritinemia, elevated sIL-2R, increased triglycerides, and hemophagocytosis observed in bone marrow analysis. Treatment with dexamethasone led to a decline in inflammatory markers and ferritin levels. Subsequently, the patient developed acute kidney failure with nephrotic syndrome, likely secondary to HLH. Additionally, a reactivation of cytomegalovirus (CMV) was observed, with a viral load of 14,000 Geq/mL. The patient discharged herself against medical advice prematurely, and the last measured viral load was 68,000 Geq/ml.

In Cologne (Table 1, Cologne_Hem), a case involved an elderly patient in their 70s (Cologne_Hem1) who presented with a painful, livid skin rash and vasculitis. High PB19V viremia was detected (2.16 million IU/mL), and a skin biopsy confirmed the presence of vasculitis triggered by viral infection. The patient responded well to corticosteroid therapy and his exanthema resolved promptly. In contrast, a younger patient in their 20s (Cologne_Hem3) with CD19 + acute lymphoblastic leukemia post-allogeneic hematopoietic cell transplant, presented with persistent pancytopenia and fever. PB19V viral load reached 27 million copies/mL. IVIG therapy eventually led to clinical stabilization and a reduction in viral load over several weeks. Interestingly, another case demonstrated that anemia is not always a leading symptom of PB19V infection. A patient in their 30s with T-lymphoblastic lymphoma (Cologne_Hem4) presented with a hemoglobin level of 12.7 g/dL and generalized exanthema. Despite persistent PB19V viremia over two months during induction treatment for lymphoma, the patient did not develop anemia.

In Magdeburg (Table 1, Magdeburg_Hem), a patient in their 30s (Magdeburg_Hem1) presented after a recent febrile upper respiratory infection. Immature granulocytes seen by the general practitioner raised concern for acute leukemia, but hematology evaluation revealed normal blood counts. A peripheral smear showed relative monocytosis and plasmacytoid lymphocytes. PB19V DNA was detected at a high level (2,911,500 IU/mL), consistent with a resolving infection; no further diagnostics or treatment were required.

A second case (Magdeburg_Hem2) involved a mid-40s patient with HS, presenting with fatigue, headache, and recent respiratory symptoms. Severe anemia (Hb 4.0 g/dL) with tachycardia was due to hemolysis from acute PB19V infection (13,423,750 IU/mL) and folate deficiency. The patient received five units of RBCs and folate supplementation. At follow-up 19 days later, the patient had clinically improved, hemoglobin rose to 10.5 g/dL, and viral load declined to 420,250 IU/mL, with residual mild hemolysis attributable to the underlying condition.

Next, we focused on children and adolescents and gathered a series of compelling cases (Table 1, Munich_Ped). One case involved a preschool-aged child with sickle cell disease (Munich_Ped1) who presented with severe anemia (Hb 5.0 g/dL), fever, and a vaso-occlusive crisis. The patient required a transfusion, and PB19V PCR revealed a significantly elevated viral load (> 100 million Geq/mL). Another middle childhood patient with DiGeorge syndrome, a T-cell immunodeficiency (Munich_Ped3), presented with fever, cervical lymphadenopathy (LAD), and mild anemia (Hb 10.7 g/dL). Despite a high viral load (> 100 million Geq/mL) and positive IgM antibodies, the patient did not require a transfusion. In this case, a lymph node biopsy was performed to rule out lymphoproliferative disorders, given the predisposition associated with Di-George syndrome and underlying immune deficiencies. Another school-aged child (Munich_Ped4) previously healthy and diagnosed with IgA vasculitis presented with purpura, fever, abdominal pain, and mild thrombocytopenia but stable hemoglobin levels (12.2 g/dL), requiring no transfusion. Elevated PB19V PCR and positive IgG and IgM antibodies confirmed the association between PB19V and vasculitis. Lastly, a pre-school aged child with iron deficiency anemia (Munich_Ped5) presented with critically low hemoglobin (Hb 2.4 g/dL), fever, and mild thrombocytopenia, and no further symptoms. The patient required a transfusion, and PB19V PCR revealed a viral load of 9 million Geq/mL, with positive IgG and IgM antibodies further complicating the clinical picture of pre-existing anemia.

**Table 1:** Overview of the diverse clinical presentations of PB19V infection in hematology and pediatrics

Site	Comorbidities	Age Group (years)	Hemo- globin [g/dL]	Transfusion (Number of RBCs)	Clinical presentation
Munich_Hem1	HS	20–30	5.9	4	Hepatitis, arthritis, gastroenterities
Munich_Hem2	HS	10–20	5.9	2	Headache, fever
Munich_Hem3	none	40–50	6.5	4	Hemophagocytic lymphohistiocytosis (HLH Criteria 7/9)
Munich_Hem4	Chronic lymphocytic leukemia (CLL)	70–80	4.5	2	Dizziness, tachycardia, fever, Evans syndrome with coombs positive hemolytic anemia
Cologne_Hem1	none	70–80	11.5	0	Pain, rash, vasculitis
Cologne_Hem2	none	30–40	6.9	6	HLH, several other infections (bacterial, fungal, viral)
Cologne_Hem3	Common B-ALL, post allogenic stem cell transplantation	20–30	7.2	2	Fever, generalized pain
Cologne_Hem4	T-lymphoblastic lymphoma	20–30	12.7	4	Generalized exanthema
Magdeburg_Hem1	none	30–40	11.5	0	Respiratory symptoms
Magdeburg_Hem2	HS	40–50	4.0	5	Fever, fatigue, respiratory symptoms, folate deficiency
Munich_Ped1	Sickle cell disease	< 5	5	1	Fever, vaso-occlusive crisis
Munich_Ped2	None	< 5	5.9	1	Fever, jaundice
Munich_Ped3	DiGeorge syndrome (partial)	5–10	10.7	0	Fever, cervical lymphadenopathy
Munich_Ped4	None	5–10	12.2	0	Purpura, fever, abdominal pain IgA vasculitis
Munich_Ped5	Iron deficiency	5–10	2.4	1	Fever, headache

Each row represents an individual patient case from the hematology and pediatric departments in Munich (Munich_Hem, Munich_Ped), as well as from the hematology department in Cologne (Cologne_Hem) and Magdeburg (Magdeburg_Hem). Columns show key aspects including, site of care, comorbidities, age group, hemoglobin levels, number of transfusions, and clinical presentation.

In the gynecology department of the LMU University Hospital, several cases highlight the severe risks associated with PB19V infection during pregnancy (Table 2, Munich_Gyn). A woman in her 30s (Munich_Gyn1) presented for first-trimester screening at 11 + 6 weeks of gestation. Although asymptomatic, she had been diagnosed with a PB19V. The scan revealed an increased nuchal translucency (5.6 mm), but the fetus appeared otherwise normal. Six days later, during a planned amniocentesis, no fetal cardiac activity was detected. The fetus was delivered at 13 + 0 weeks following miscarriage induction. Another case involved a woman in her 20s at 28 + 4 weeks of gestation (Munich_Gyn2), who experienced intrauterine fetal death, presenting with absent fetal movements. A recent PB19V infection, likely contracted before 20 weeks of pregnancy, was identified. Despite no abnormalities observed on autopsy or placental histology, significant fetal anemia caused by the infection was suspected as the primary cause of death. Case Munich_Gyn4 illustrates a favorable outcome with the birth of a healthy newborn following successful intrauterine blood transfusions. Nevertheless, it is crucial to consider the implications of PB19V infection in younger populations. Children under three years of age demonstrate low immunity to PB19V and many infected children remain asymptomatic. The rising number of infections places seronegative pregnant women at increased risk, particularly those with young children in their household (3).

**Table 2:** Overview of the diverse clinical presentations of PB19V infection in pregnant women

Site	Week of gestation at first presentation	Clinical presentation (ultrasound)	Outcome
Munich_Gyn1	10	Widened nuchal translucency	CVS planned, but IFD WG 12 + 4
Munich_Gyn2	28	Absent fetal movement PB19V high avidity (first tested after IFD)	Intrauterine blood transfusion was planned, IFD WG 28 before puncture.
Munich_Gyn3	unknown	Increased flow velocity of the middle cerebral artery, pericardial effusion.	IFD WG 16
Munich_Gyn4	19	Increased flow velocity of the middle cerebral artery	Healthy mature newborn, intrauterine blood transfusion twice

Each row represents an individual patient case. Columns present key aspects such as site of care, week of gestation at first presentation, clinical presentation assessed via ultrasound, and pregnancy outcome. CVS = chorionic villus sampling, IFD = intrauterine fetal death, WG = week of gestation

### Virological analyses

**Fig. 3:**
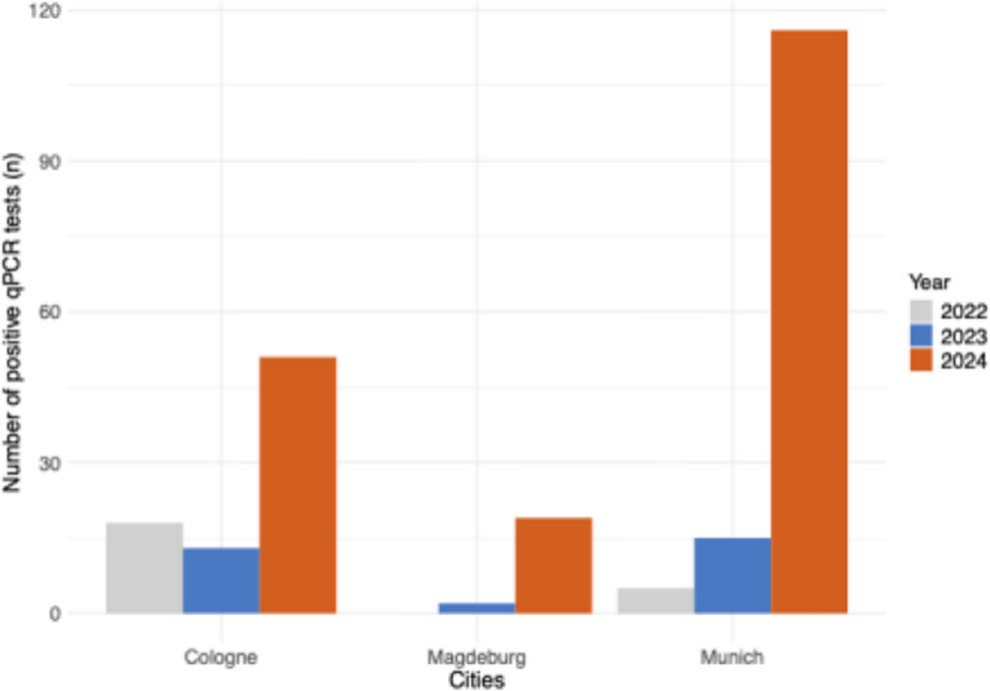
Total number of positive PCR test results (from any material) from 2022 to 2024 across three sites: Cologne, Magdeburg, and Munich. Only one positive test per patient was counted

**Fig. 4:**
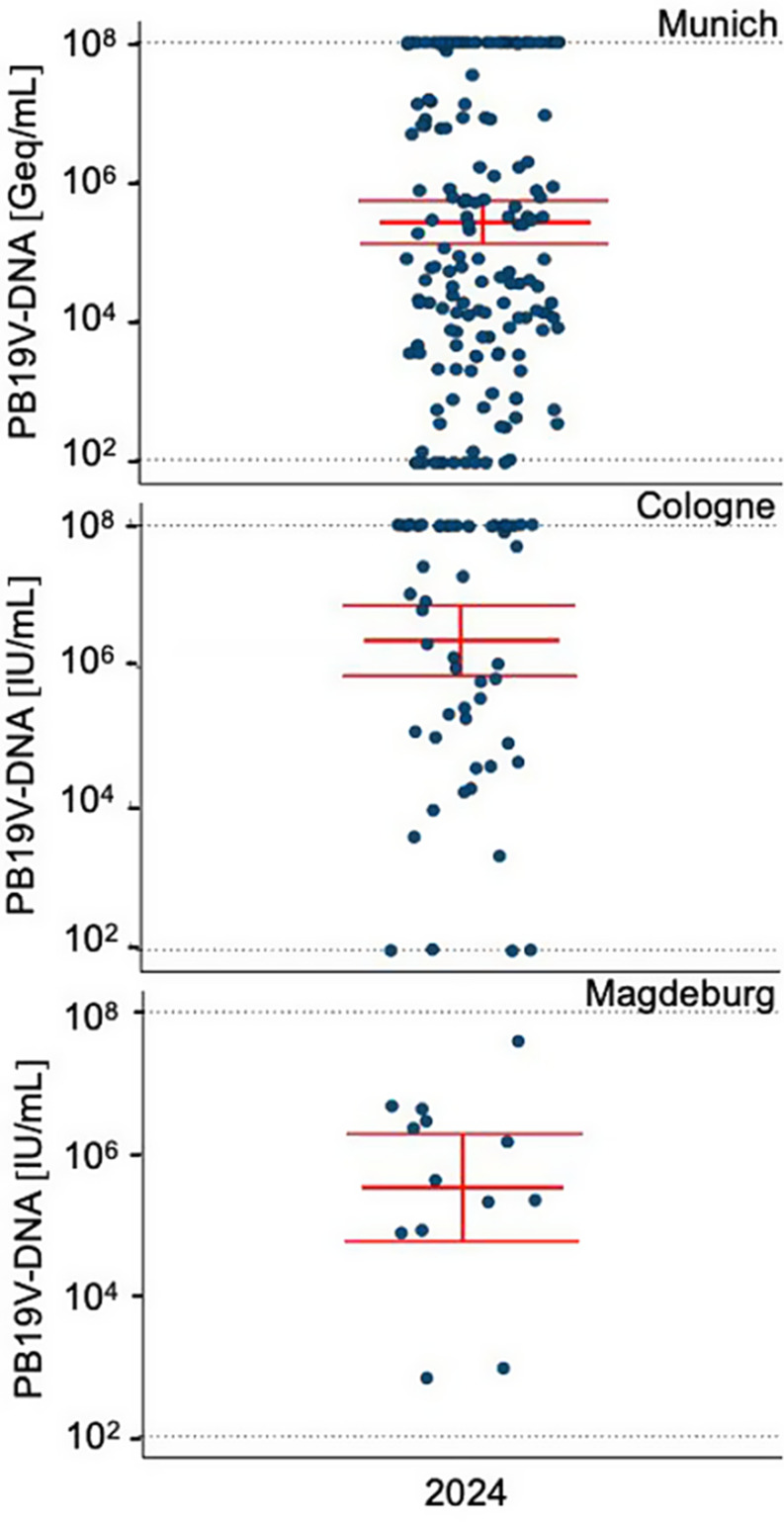
Each dot represents the highest measured PB19V DNA level for an individual PCR-positive patient, all measured in blood samples. Data from Munich (*n* = 116) are shown in Geq/mL, while data from Cologne (*n* = 56) and Magdeburg (*n* = 10) are presented in IU/mL. Red horizontal lines indicate the geometric mean, and vertical red bars show the 95% confidence interval. Dotted black lines represent the lower detection limit (10^2^) and the upper saturation threshold (10^8^)

**Fig. 5:**
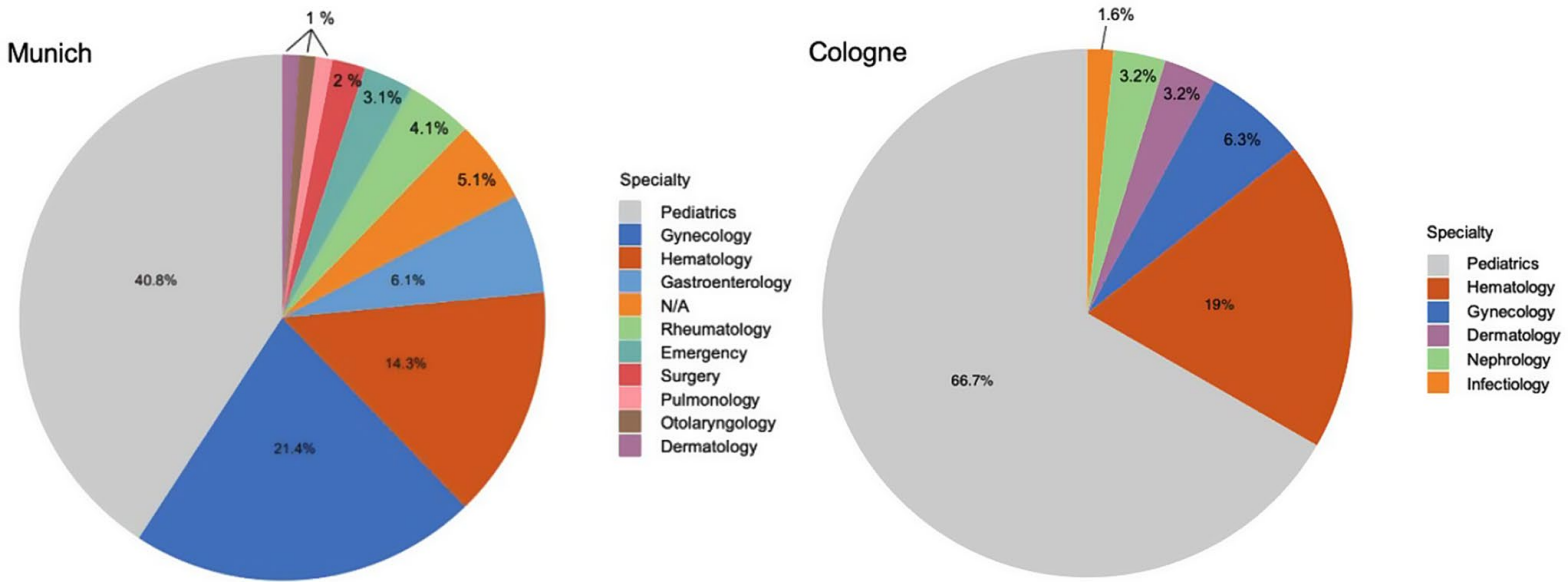
Pie chart displaying the distribution of PB19V PCR test submissions by medical specialty at the LMU University Hospital and the Cologne University Hospital. Submitting departments include pediatrics, gynecology, hematology, gastroenterology, rheumatology, emergency, surgery, pulmonology, otolaryngology, dermatology, and cases categorized as not applicable (N/A)

As in most European Union countries, PB19V infections are not subject to systemic surveillance in Germany. There is no nationwide reporting obligation under the Infection Protection Act (IfSG), resulting in a lack of epidemiological data on incidence and seroprevalence. To address this gap and improve data visibility, we present virological data from Munich, provided by the Max von Pettenkofer Institute (MvP), and from Cologne and Magdeburg contributed by their respective virology departments. Between January and December 2024, a total of 936 qPCR tests for PB19V were conducted by the MvP. Strikingly, the number of positive cases increased markedly, with the first quarter of 2024 alone exceeding the total annual number of positives recorded in previous years. Specifically, 48 PCR tests returned positive results in the first quarter, corresponding to a patient-based positivity rate of 16%, followed by a rate of 18.2% in the second quarter. A sharp decline in positivity was observed in the third and fourth quarters, dropping to 1.2%. For comparison, in 2023, only 15 of 641 PCR tests were positive for PB19V, yielding a positivity rate of 2.3%. In the first quarter of 2025, we observe an even lower positivity rate of 0.8%, reflecting reduced transmissions following prior population exposure. However, factors such as seasonal variation and reduced testing intensity post-peak may also contribute to this trend, and definitive conclusions regarding herd immunity require supporting seroprevalence data. Interpretations remain challenging, as individual infection courses are largely unavailable. Notably, the overall testing volume per quarter tripled in 2024 compared to the previous year. This upward trend in PB19V detection is further supported by virological data from the University Hospitals of Cologne and Magdeburg (Fig. 3 Number of positive PB19V PCR tests (total numbers) per medical specialty in Munich). At all three centers, most infections were acute and characterized by high viral loads (Fig. 4 PB19V-DNA peak levels in positive patients during 2024 in Munich, Cologne, and Magdeburg). The majority of test submissions originated from pediatrics (40.8–66.7%), followed by hematology. At Otto von Guericke University Magdeburg, all submitted cases were from the hematology department. At LMU University Hospital Munich, a notable number of cases originated from gynecology (21.4%). This distribution reinforces the identification of particularly vulnerable populations affected by PB19V. Additionally, departments such as gastroenterology and nephrology/rheumatology also contributed a significant number of positive cases, suggesting that PB19V may play a role in complicating conditions in these specialties. Smaller numbers of cases were reported from dermatology, otolaryngology, infectiology, and pulmonology, indicating that while PB19V may be less frequently encountered in these fields, it still presents in a broad range of clinical scenarios (Fig. 5 Number of positive PB19V PCR tests (total numbers) per medical specialty in Munich, Cologne, and Magdeburg).

## Discussion

The findings of this study highlight a significant rise in PB19V infections across multiple centers and medical specialties in Germany in 2024, a trend that aligns with similar reports from other countries, such as Israel, the Netherlands, and France in 2023 and 2024 [7–9]. Although sequencing was not routinely performed at our centers, reports from other European regions indicate that genotype 1a, the most common of the three known PB19V genotypes in Europe, continues to predominate among circulating strains [10]. These findings suggest a broader pattern of increased PB19V circulation rather than isolated outbreaks. A likely contributing factor are the effects of the COVID-19 pandemic, which disrupted typical viral transmission patterns as seen with other pathogens. Changes in healthcare utilization, social behaviors during so-called lockdowns, and shifts in immune responses may have contributed to fluctuations in levels of exposure and susceptibility to PB19V as seen in the Netherlands [9]. The demographic distribution remains consistent with prior years, with the highest burden in children and women of childbearing age. Notably, recent cases from LMU Munich’s gynecology department involved miscarriage and fetal complications during early pregnancy. In the absence of antiviral therapy or vaccines, early detection and close monitoring remain critical. Clinically, PB19V testing should be considered in patients with unexplained anemia, pancytopenia, or fever, especially in hematologic and immunocompromised populations. In conclusion, the significant rise in symptomatic PB19V infections in 2024 underscores the need for heightened clinical awareness and screening protocols, particularly among high-risk populations, helping to mitigate the overall burden of disease. However, as immunity increases in the aftermath of this wave, the number of susceptible individuals is expected to decline. Based on epidemiological observations from previous cycles the next substantial resurgence of PB19V may not occur for another four to six years [2]. These findings highlight the immediate need for clinical vigilance but also the importance of long-term monitoring strategies to anticipate future outbreaks and protect vulnerable groups.

## Data Availability

No datasets were generated or analysed during the current study.
